# Taxonomic and functional responses of benthic and drifting macroinvertebrates to fine sediment deposition: evidence from an alpine flume-based experiment

**DOI:** 10.1007/s10750-025-06014-w

**Published:** 2025-11-13

**Authors:** Alberto Doretto, Andrea Faiola, Serena Masserano, Paola Rossi, Matteo Sassone, Matteo Zanarotto, Camilla Zucchi, Paul J. Wood, Kate L. Mathers

**Affiliations:** 1 Department for the Sustainable Development and Ecological Transition, University of Eastern Piedmont, Piazza Sant’Eusebio 5, 13100 Vercelli, Italy; 2 Alpine Stream Research Center/ALPSTREAM, 12030 Ostana, Italy; 3 Geography and Environment, Loughborough University, Loughborough, Leicestershire LE11 3TU UK

**Keywords:** Diversity, River ecology, Siltation, Mesocosm, Interstitial, Alps

## Abstract

Excessive fine sediment deposition is a global pervasive issue in rivers and, in particular, for benthic organisms including macroinvertebrates. However, the assessment of fine sediment effects on the taxonomic and functional diversity of macroinvertebrate communities is challenging because of context-dependent confounding factors, such as river-flow conditions, land-use change, and anthropogenic pressures. By using a set of outdoor artificial flumes, this study examined the immediate (24–48 h) and two weeks post-addition response of Alpine stream macroinvertebrates to experimental deposition of fine sediment (7.5 kg m^2^). The taxonomic composition of benthic macroinvertebrate communities demonstrated significant differences between control and sediment-affected flumes; while taxonomic composition of drifting macroinvertebrates varied over time, with higher taxonomic heterogeneity for sedimented communities than control flumes. Nestedness was consistently the dominant component that contributed to beta-diversity patterns for both benthic and drifting invertebrates. No differences in taxonomic and functional community metrics were observed between control and sedimented communities, with the exception of a significant reduction in the total abundance of benthic macroinvertebrates in sedimented flumes. This study is one of the first flume-based experiments dealing with the response of Alpine stream macroinvertebrates to fine sediment deposition, and the findings highlight the context specific nature of fine sediment effects.

**Supplementary Information:**

The online version contains supplementary material available at 10.1007/s10750-025-06014-w.

## Introduction

Fine sediment is commonly defined as particles < 2 mm in diameter (Wood & Armitage, 1997; Harper et al. 2017). Although a natural component of riverine sediment regimes, a variety of anthropogenic activities often deliver an excessive volume of fine sediment to rivers through surface runoff which may potentially increase in the future due to changes in rainfall and runoff regimes (Burt et al. 2016). These activities include mining (Smolders et al. 2003; Bona et al. 2016), deforestation (Betts et al. 2022), and intensive agriculture (Sutherland et al. 2012; Naden et al. 2016). Additionally, activities such as reservoir sediment dredging/flushing operations (Quadroni et al. 2023, 2024) and reduced hydrological variability associated with damming represent anthropogenic activities that typically lead to increased infiltration and instream storage of fine sediment within the riverbed (Sear, 1993; Mathers et al. 2021). Several terms, including siltation, clogging, and riverbed colmation, have been specifically adopted to denote such phenomenon and its related consequences (Wharton et al. 2017). Based on a systematic search using the keywords “Siltation OR Clogging OR Riverbed colmation AND River” on the Web of Science database (search carried out on 16 January 2025), a total of 17,024 publications across all the main fields of research were found. The number of papers has increased over time from 236 articles in 2001 to 1,272 in 2024, highlighting the growing prominence of fine sediment-related issues that are being recognized more widely as a source of hydro-morphological alteration for river ecosystems globally, which often leads to reductions in biodiversity (Owens et al. 2005; McKenzie et al. 2024).

Instream sedimentation has been shown to have significant implications for the entire lotic food web (Kemp et al. 2011; Jones et al. 2012a; 2014). Enhanced deposition on the riverbed can lead to infilling of the interstitial pore space between substrate particles (i.e. streambed clogging), and this, in turn, typically leads to the loss and homogenization of habitats, in addition to reductions in intra-gravel dissolved oxygen levels (Bo et al. 2007; Larsen et al. 2011; Wharton et al. 2017). Furthermore, the formation of surficial fine sediment deposits has indirect impacts on the availability and quality of energetic inputs. Previous studies have shown that river reaches characterized by an excessive accumulation of fine sediment usually display a reduced amount of coarse particulate organic matter (CPOM), as a consequence of the loss of the substrate retention capacity, as well as a reduction in primary production associated with burial by sediments (Kreutzweiser et al. 2005; Doretto et al. 2016).

Spending a significant part, if not all of their life, in contact with the substrate, benthic macroinvertebrates demonstrate a high sensitivity to changes in substrate conditions (Jones et al. 2012b; Hubler et al. 2016). They constitute a heterogeneous group of taxa with a wide range of sensitivities to the additive and synergistic effects of fine sediment and other abiotic conditions within river ecosystems, including water chemistry and flow conditions (Wood & Armitage, 1999; Archaimbault et al. 2010; Benoy et al. 2012; Magbanua et al. 2013; Pastorino et al. 2020). Thus, benthic macroinvertebrates represent excellent bioindicators to evaluate the implications of fine sediment, with studies documenting community-level changes in taxonomic and functional composition in relation to the short- and long-term effects of fine sediment (Angradi, 1999; Murphy et al. 2015; Doretto et al. 2018a; Gieswein et al. 2019; Mathers et al. 2022). In addition, higher drift rates have been reported as an immediate response of macroinvertebrates to excessive fine sediment addition and transport (Suren & Jowett, 2001; Larsen & Ormerod, 2010; Larsen et al. 2011). Beyond being one of the main dispersal mechanisms of riverine macroinvertebrates, drift is often adopted by these organisms to evade adverse environmental conditions, or as an immediate response to sudden changes in the surrounding habitat, such as sediment pulses (Milner et al. 2021). Although some taxa demonstrate a tolerance to increasing levels of fine sediment (e.g. Chironomidae and Oligochaeta), excessive fine sediment deposition generally leads to biodiversity loss and reductions in macroinvertebrate abundance with increasing clogging excluding EPT (Ephemeroptera, Plecoptera, and Trichoptera) taxa and macroinvertebrates with a larger body size, external gills, and specialized feeding strategies, e.g. scrapers, shredders, and filter feeders (Rabení et al. 2005; Couceiro et al. 2011; Wilkes et al. 2017).

Although there is a large body of scientific literature on the response of macroinvertebrates to excessive fine sedimentation, contrasting results have been widely observed (Wilkes et al. 2017) and this is likely due to the difficulties of disentangling the direct effect of fine sediment from those associated with other co-occurring factors in field studies. For instance, the response of macroinvertebrates to siltation is often conducted in agricultural streams and rivers, yet, in these settings, potential additive impacts often originate from chemical impairment due to the occurrence of pesticides and fertilizers (Wagenhoff et al. 2011, 2017; Burdon et al. 2013). The geology, hydrology, and land use of the watershed also have a strong influence on sediment dynamics and its seasonal variation, thus making difficult to make direct comparisons between different rivers and studies (Larsen et al. 2009; Buendia et al. 2011; Bylak & Kukuła, 2022; Davis et al. 2022). In the light of this, the use of mesocosms, especially those in the form of in situ artificial flumes that simulate a river reach, can represent a valid experimental and manipulative approach to study the response of macroinvertebrates to fine sediment by controlling key environmental parameters, such as water quality, discharge, and the type of fine sediment (Lamberti & Steinman, 1993; Turunen et al. 2018; Menczelesz et al. 2020). Owing to their versatility, flume-based experiments in the field of river ecology have blossomed in recent decades allowing assessment of the ecological consequences of different instream disturbances, including drought (Aspin et al. 2019), light pollution (Juvigny-Khenafou et al. 2024), and flow alterations (Bækkelie et al. 2017; Vallefuoco et al. 2022).

In this study, a set of outdoor artificial flumes were used to elucidate the direct response of Alpine stream macroinvertebrate communities to fine sediment addition. Compared to most previous studies, this research simultaneously examined the impacts of fine sediment addition and deposition on both the benthic invertebrates (i.e. substrate-associated) and those entering into drift (hereafter termed drift communities). By adopting a manipulative approach, changes in alpha and beta diversity as well as the functional diversity of macroinvertebrate communities could be directly examined in a semi-controlled environment, thereby avoiding potential biases associated with current velocity, water chemistry and land use. We expected enhanced drift density and differences in the taxonomic composition of drifting macroinvertebrate communities as a short-term consequence of fine sediment addition. We also hypothesized that fine sediment deposition would modify the substrate conditions for benthic macroinvertebrates with the following consequences: i) distinct differences in the taxonomic composition of control and sediment-affected communities, ii) increased contribution of nestedness (i.e. taxa gain/loss) to beta diversity compared to control communities; iii) reduced taxonomic richness, total abundance, and functional diversity in sedimented flumes.

## Materials and methods

### Experimental design and data collection

**Fig. 1:**
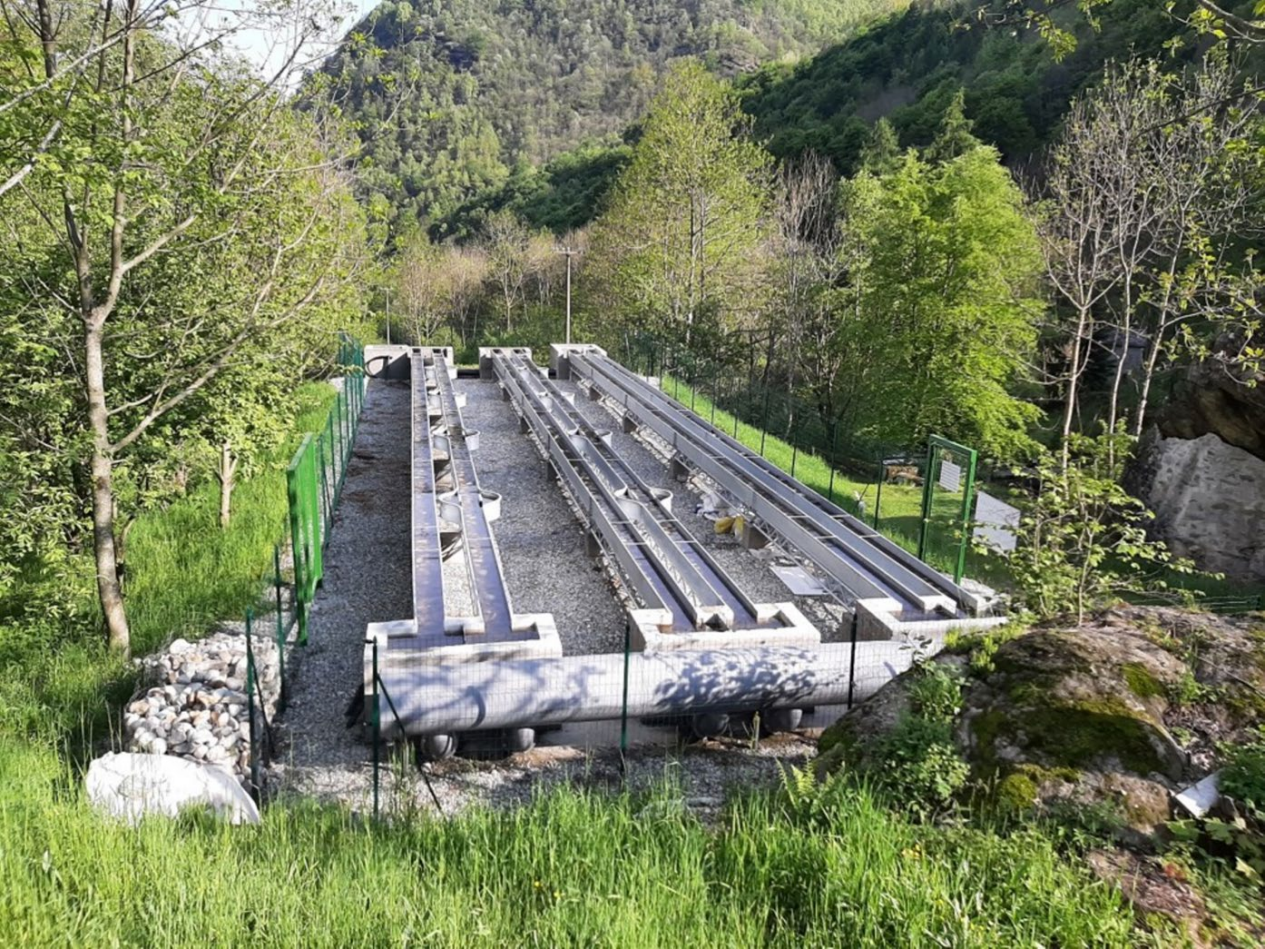
Outdoor artificial flumes in Ostana (Italy) used in the experiment. Flume n.1 is on the right, while flume n.6 is on the left. Flumes are fed by the Po River via a water diversion located around 50m upstream

The experiment was performed using the set of outdoor artificial flumes of the Alpine Stream Research Center/ALPSTREAM in Ostana (Northwestern Italy, Cottian Alps, Cuneo Province). This mesocosm facility is located on the left bank of the Po River and consists of six 25 m long, 0.30 m wide, and 0.30 m deep metal flumes that are directly fed by the Po River (elevation: 1,119 m a.s.l.; 44°41′33.88′′ N; 7°10′38.33′′ E). Flumes are grouped in three blocks and are numbered from *n*.1 to *n*.6, with flume *n*.1 being the nearest to the Po River bank (Fig. 1). A volume of water (maximum 50 L/s) is diverted from the Po River, around 50 m upstream, and delivered to the flumes through a pipe (diameter = 60 cm). At the entrance of the pipe there is a large-sized metallic grid to capture large wood and other debris. Water is continuously able to flow from the Po River to the flumes via gravity when the inlets of the flume are open (Supplementary Information Fig. S1). The design and size of these flumes are similar to those used in previous research (Ledger et al. 2012; Lancaster & Ledger, 2015), including flume-based experimentation in the Alps (Bruno et al. 2016; Grubisic et al. 2017; Doretto et al. 2018b; Gruppuso et al. 2021).

The experimental setup (T0) took place on 17 and 18 April 2023. In each flume, the same quantity of quarry-derived gravel, cobbles, and coarse sand was manually added to the flumes to reproduce the substrate composition of the Po River. Flume inlets were opened on 18 April, thus enabling drifting macroinvertebrates to colonize all flumes. Additionally, similar to previous studies (Milner et al. 2022; Burgazzi et al. 2024) to facilitate colonization, from 19 to 26 April, macroinvertebrates were seeded by adding 15 Surber samples to each flume (area = 0.05 m^2^ each sample) collected from the Po River by the same operator (15 Surber samples X 6 flumes = 90 Surber samples). To avoid biases associated with the taxon-specific preference of macroinvertebrates to substrate, Surber samples were collected on comparable mineral substrates (i.e. cobbles and gravel) that occurred in the flumes.

Following three weeks of colonization, the experiment began on the 8 May 2023. For each of the three flume blocks, one flume was selected as a control (i.e. flumes *n*.2, *n*.4, and *n*. 6) and one flume as the “impact” which underwent sediment addition (i.e. flumes *n*.1, *n*.3, and *n*.5). A total of 7.5 kg m^2^ of sterile play sand (diameter = 0.5–1.5 mm), to ensure no confounding influence of turbidity from finer fractions, was added across the entire impacted flumes. Play sand was slowly added to the flume by hand ensuring consistent coverage across the entire flume and was applied from upstream to downstream. Invertebrate sampling took place on two occasions: following sediment addition (24 h for drift and over a time span of 48 h for benthic)—hereafter termed T1, and 2 weeks following sediment addition—hereafter T2. On each occasion, drifting and benthic invertebrates were sampled.

Drifting invertebrates were sampled by installing one drift net (25 × 25 cm; mesh size = 250 µm) at the end of each flume (*N* = 6 per time point) for 24 h. Water depth and velocity in front of each drift net was measured to ensure there was no variation in hydraulic factors influencing drift between the flumes. Drift nets were installed prior to sediment addition to capture immediate responses to fine sediment addition. Benthic invertebrates were sampled by taking six Surber samples (23 × 22 cm, 0.05 m^2^, mesh size: 0.5 mm; Doretto et al. 2020) in each flume starting downstream and working upstream (*N* = 36 per time point). All collected macroinvertebrates were preserved in 94% ethanol in the field. A total of 12 drift samples and 72 Surber samples were collected over the two sampling campaigns. Samples were returned to the laboratory where macroinvertebrates were sorted, counted, and taxonomically identified to the genus (mostly Plecoptera, Ephemeroptera) or family level by using a stereomicroscope (Leica, mod. EZ4), and the taxonomic keys for Italian fauna (Campaioli et al. 1994; 1999).

To ensure water quality did not vary over the course of the experimental period, water temperature, pH, electrical conductivity, and dissolved oxygen concentrations were measured in the Po River with a multiparametric probe (Hanna, mod. HI98194) on all sampling occasions (T0 to T2).

### Statistical analyses

The same statistical analyses (significance threshold: *p*-value < 0.05) were performed on the drift and benthic data in the R environment (R Core Team, 2024; Wickham et al. 2024). Given that only one sample was obtained for the drift community of each flume (*N* = 6), all Surber samples were pooled for each flume to provide a composite macroinvertebrate community (*N* = 6). This also avoided possible confounding biases in the response of macroinvertebrate abundance and occurrence due to small-scale substrate patchiness inside the flumes associated with the small sample area (23 × 22 cm).

#### Compositional differences between sediment treatments

Based on the abundance of macroinvertebrate taxa, clamtest analysis (clamtest function, “vegan” R package—Oksanen et al. 2015) was first applied to identify indicator taxa for the control and impacted flumes. By applying a multinomial model on the relative abundance of taxa in the two different treatments, this analysis classifies them as specialists or generalists (Chazdon et al. 2011). Differences in the taxonomic composition of the benthic and drifting macroinvertebrate communities were visualized using principal coordinate analysis (PCoA), with permutational analysis of variance (PERMANOVA) applied to statistically test for significant differences associated with the factors of treatment, time and their interaction on macroinvertebrate community composition (adonis2 function, “vegan” R package—Oksanen et al. 2015). To test for differences in the heterogeneity of taxonomic composition of benthic and drift macroinvertebrate communities by sediment treatment, the test of homogeneity for multivariate dispersion was run (PERMDISP; Anderson, 2006; betadisper function in “vegan” R package—Oksanen et al. 2015). Bray–Curtis dissimilarity indices were used as distance measures in all multivariate analyses. To evaluate the taxa contributing to any compositional changes in the benthic and drift macroinvertebrate communities by time and treatment, similarity percentage (SIMPER) analysis was performed (simper function, “vegan” R package—Oksanen et al. 2015).

#### Patterns in beta diversity and its components

To further investigate the mechanisms of compositional changes in benthic and drifting macroinvertebrate communities, total beta diversity and its nestedness (i.e. loss/gain of taxa) and turnover (i.e. taxa replacement) components were calculated (beta.multi function, “BAT” R package—Cardoso et al. 2022) for each pairwise comparison by time and treatment following Baselga (2010). This analysis allowed patterns in beta diversity and its components between sediment-affected and control macroinvertebrate communities to be highlighted as well as temporal differences for each treatment community over time.

#### Differences in taxonomic and functional diversity between sediment treatments

Total taxon richness and total macroinvertebrate density were calculated for each flume for the benthic dataset, while the total drift richness and total drift density were obtained from the drift samples. Total drift density was calculated as the number of macroinvertebrates per volume of water (N.Individuals/100m^3^) following the approach of Hauer & Lamberti (2017). Functional traits were assigned to each macroinvertebrate taxon according to the classification of Tachet et al. (2010), and for both datasets, the following functional metrics were calculated: functional richness, functional evenness, and functional dispersion (“biomonitoR” R package; Laini et al. 2022). Functional richness was calculated as the amount of functional space filled by the community, while functional evenness represents how evenly the abundance of taxa is distributed in the functional trait space (Villéger et al. 2008). Functional dispersion quantifies the spread of the taxa in the trait dimensional space (Laliberte & Legendre, 2010). Statistical differences in the taxonomic and functional metrics between control and sediment-affected flumes were tested by applying Generalized Linear Models (GLMs) or Linear Models (LMs) using the glm and lm functions in R (R Core Team, 2024), respectively. Taxonomic and functional metrics were included as response variables in the regression models, with the fixed interacting effects of “Time” (i.e. T1 vs T2) and “Treatment” (i.e. control vs sediment-affected flumes). GLMs with Poisson or negative binomial distributions were used for count data, while LMs with logit-transformed data (logit function in “car” R package; Fox et al. 2024) were used for proportional data.

## Results

No influential changes in the physical and chemical parameters of water were recorded throughout the experiment, despite a progressive decrease in the electrical conductivity from 178 to 88 µs/cm (Table S1). The average water velocity in the control and sedimented flumes ranged between 0.12 and 0.18 m/s, while the average water depth varied from 9.67 to 11.17 cm (Table S1).

A total of 11,552 macroinvertebrates belonging to 35 different taxa were recorded in the benthic dataset (Table S2). Chironomidae (88%) were the most abundant taxon, followed by *Amphinemura* sp. (5.5%) and *Baetis* sp. (1.8%). The average number of taxa and individuals per sample were 7 (± 2.49 SD) and 162 (± 75.11 SD), respectively. Based on their abundance and according to the clamtest analysis, six taxa were classified as sediment specialists: *Protonemura* sp., *Amphinemura* sp., Hydropsychidae, Elmidae, Empididae, and Psychodidae, while three taxa were classified as specialists for the control treatment: *Nemoura* sp., *Habroleptoides* sp., and Chironomidae. All remaining taxa were considered generalists or too rare. The drifting macroinvertebrate community comprised 1,076 individuals belonging to 18 different taxa. Among these, Chironomidae (84%) were the most abundant taxon, followed by Lumbriculidae (7%), Nematoda (3%), and Psychodidae (2%). On average, there were five macroinvertebrate taxa per drift sample. Only *Protonemura* sp. were identified as a specialist taxon for the drifting community in the control treatment with no taxa identified in the sediment treatment.

### Compositional differences between sediment treatments

**Fig. 2:**
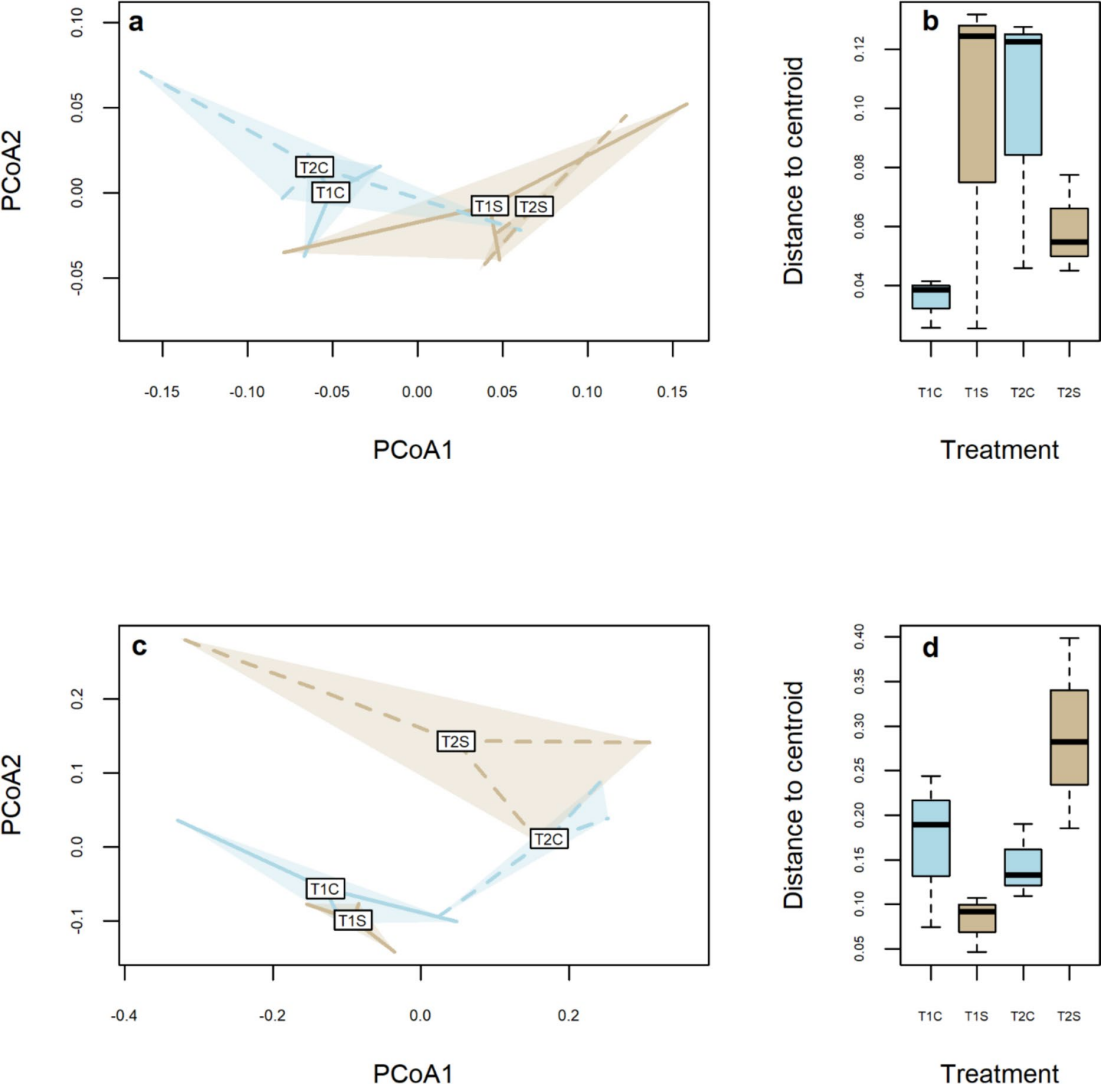
Output of the multivariate analyses: **a** Principal coordinates analysis (PCoA) plot for the benthic macroinvertebrate community; **b** boxplot illustrating the distance to centroid of the benthic macroinvertebrate communities; **c** PCoA plot for the drifting macroinvertebrate community; **d** boxplot illustrating the distance to the centroid of the drifting macroinvertebrates. T1S = sedimented macroinvertebrate communities at T1; T1C = control macroinvertebrate communities at T1; T2S = sedimented macroinvertebrate communities at T2; T2C = control macroinvertebrate communities at T2. For the boxplot: black horizontal line = median; upper and lower box edges = 3rd (75th percentile) and 1st (25th percentile) quartile, respectively; whiskers indicate ± 1.5 interquartile distance

Considering the taxonomic composition of the benthic macroinvertebrate communities (Fig. 2a), significant differences associated with the sediment treatment were observed (PERMANOVA: *F*_*1*,8_ = 3.826; *p* = 0.048), while no statistical differences were observed associated with time (*F*_1,8_ = 0.886; *p* = 0.411) or the interaction with treatment (*F*_1,8_ = 0.304; *p* = 0.794). Macroinvertebrate communities in the control and sedimented flumes displayed opposite patterns in taxonomic homogeneity over time (i.e. measured as the distance from the centroid); however, these differences were not statistically significant. Control benthic macroinvertebrate communities were more homogeneous (i.e. lower distance from the centroid) compared to the sediment treatment at T1 (Fig. 2b). In contrast, the opposite trend was observed at T2 with the control macroinvertebrate communities displaying higher heterogeneity (i.e. higher distance from the centroid) than the sediment-affected ones (Fig. 2b). Nine taxa significantly contributed to the benthic taxonomic compositional differences (Table 1). Among these, seven were from EPT orders (Ephemeroptera, Plecoptera, and Trichoptera), with the two others being Psychodidae and Naididae.

**Table 1:** Summary output of the SIMPER analysis. Only significant taxa are shown, and changes in their abundance according to the pairwise comparison are provided in parentheses ( ±). T1 Sed = sedimented macroinvertebrate communities at T1; T1 Con = control macroinvertebrate communities at T1; T2 Sed = sedimented macroinvertebrate communities at T2; T2 Con = control macroinvertebrate communities at T2

Dataset	Pairwise comparison	Taxon	*p*-value
Benthic	T1 Con versus T1 Sed	Rhyacophilidae (−)	0.006
	T1 Con versus T2 Con	Rhyacophilidae (−)	0.016
	T1 Con versus T2 Sed	Psychodidae (+)	0.044
		*Protonemura* sp. (+)	0.005
		*Serratella* sp. (+)	0.010
		Rhyacophilidae (−)	0.036
		Naididae (−)	0.048
	T1 Sed versus T2 Con	*Nemoura* sp. (+)	0.025
	T1 Sed versus T2 Sed	*Leuctra* sp. (−)	0.017
		*Protonemura* sp. (+)	0.015
		*Serratella* sp. (+)	0.001
		Psychodidae (+)	0.047
	T2 Con versus T2 Sed	*Nemoura* sp. (−)	0.048
Drift	T1 Sed versus T1 Con	*Protonemura* sp. (+)	0.020
	T1 Sed versus T2 Sed	Lumbriculidae (+)	0.042
		Lumbricidae (+)	0.016
	T1 Con versus T2 Sed	Lumbriculidae (+)	0.036
		*Protonemura* sp. (−)	0.009
		Lumbricidae (+)	0.005
		Rhyacophilidae (+)	0.021

Taxonomic composition of the drifting invertebrates varied significantly only over time (PERMANOVA: *F*_1,8_ = 3.804; *p* = 0.038) but not by sediment treatment (*F*_1,8_ = 0.358; *p* = 0.748) or the interaction of these two factors (*F*_1,8_ = 0.847; *p* = 0.468) (Fig. 2c). Taxonomic heterogeneity of drifting macroinvertebrate communities varied over time: significantly increasing from T1 to T2 in the sediment-affected flumes, while a weak decrease was observed in the control flumes but this was not statistically different (Fig. 2d). Four macroinvertebrate taxa, *Protonemura* sp., Lumbricidae, Lumbriculidae, and Rhyacophilidae, contributed to the compositional changes of the drifting invertebrate communities (Table 1).

### Patterns in beta diversity and its components

**Fig. 3:**
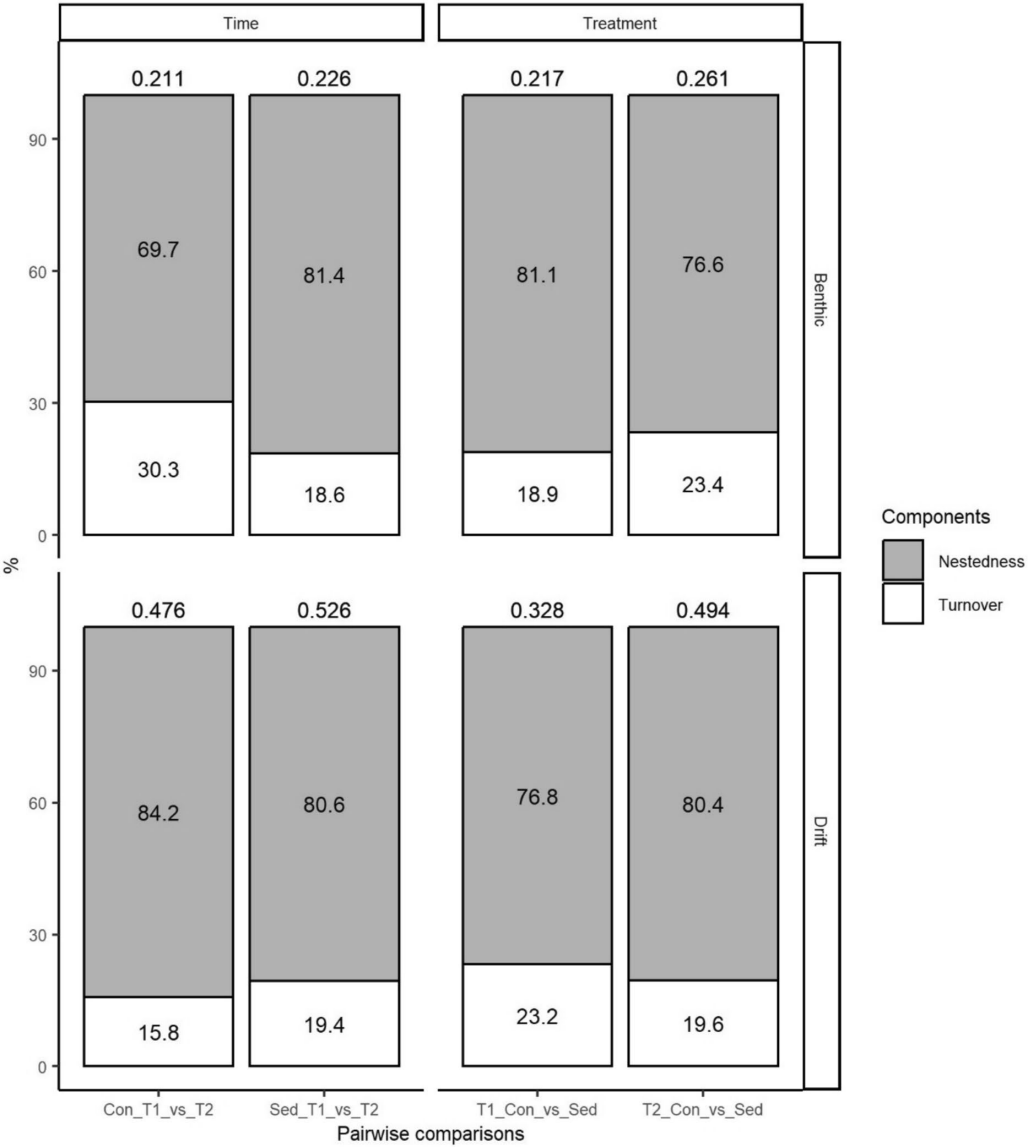
Stacked barplots displaying the average percentage contribution of nestedness (i.e. gain/loss of taxa) and turnover (i.e. taxa replacement) to total beta diversity. Numbers above the bars indicate the total beta diversity for each pairwise comparison (Con_T1_vs_T2 = comparison between the control communities of T1 and T2; Sed_T1_vs_T2 = comparison between the sedimented communities at T1 and T2; T1_Con_vs_Sed = comparison between the control and sedimented communities at T1; T2_Con_vs_Sed = comparison between the control and sedimented communities at T2). Top is benthic communities and bottom is drifting invertebrates

Total beta diversity among benthic macroinvertebrate communities ranged from 0.211 to 0.261 and was largely explained by nestedness (i.e. gain/loss of taxa) (Fig. 3). The percentage contribution of nestedness between control and sedimented communities slightly decreased from 81.1% at T1 to 76.6% at T2 associated with an increased contribution of taxa replacement (i.e. turnover) over time. Turnover contributed 30.3% to the total beta diversity between control communities from T1 to T2, while nestedness accounted for 69.7% (Fig. 3). In contrast, beta diversity between sedimented communities from T1 to T2 was mostly due to nestedness (81.4%) with turnover playing a minor role (Fig. 3).

Total beta diversity among drifting invertebrates was, on average, higher than in benthic communities, ranging from 0.328 to 0.526 (Fig. 3). Similarly, nestedness was the dominant component varying from 76.8% between control and sedimented communities at T1 to 84.2% between control communities over time. Turnover always contributed a minor component (15.8–23.2%) to the total beta diversity of drifting invertebrates (Fig. 3).

### Patterns in taxonomic and functional diversity

**Fig. 4:**
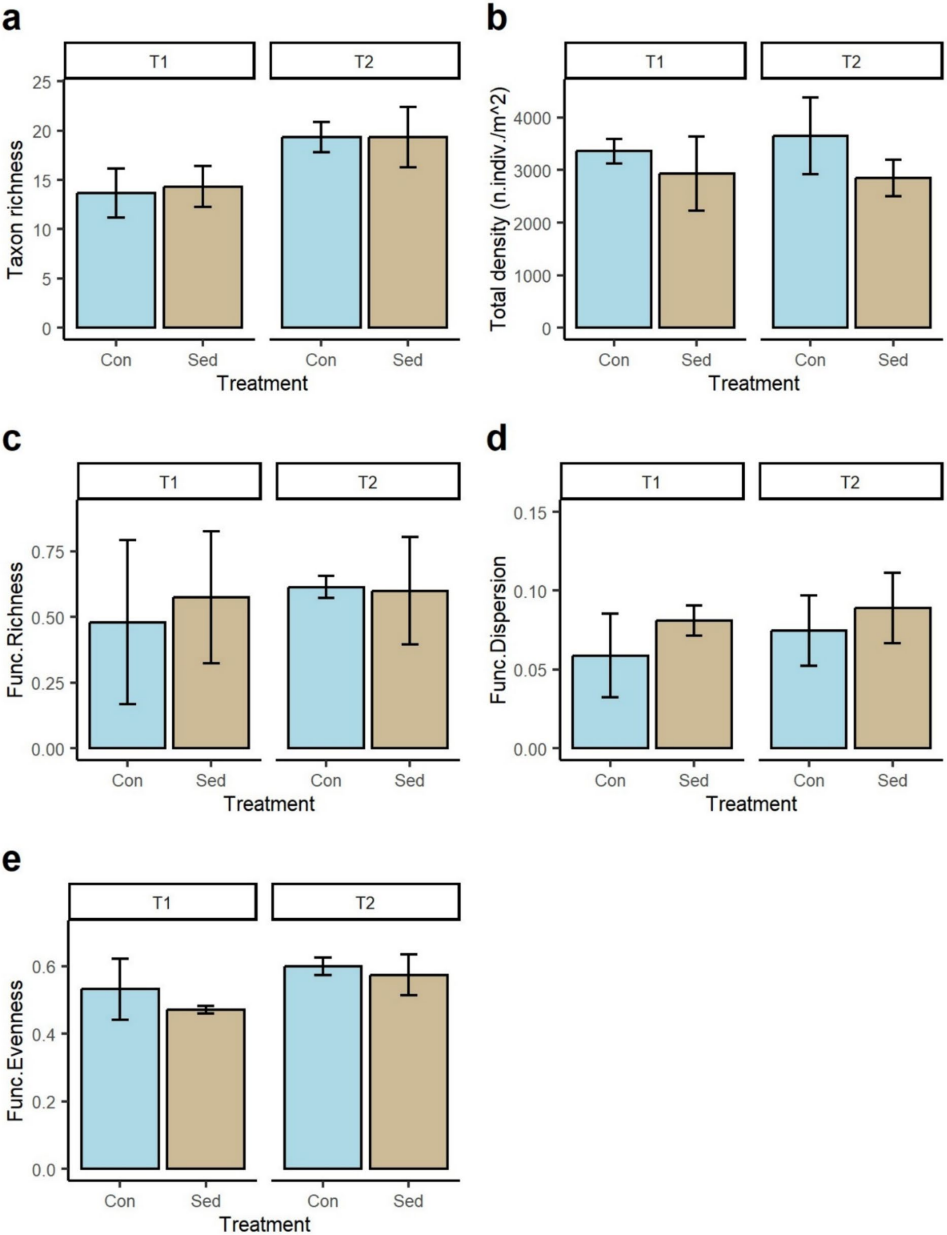
Error bars (± 1 SD) showing mean **a** taxon richness, **b** total density (number of individuals/m^2^), **c** functional richness, **d** functional dispersion, and **e** functional evenness of the benthic macroinvertebrate communities by sediment treatment on each sampling occasion. T1 = 24–48 h following sediment addition, and T2 = 2 weeks following sediment addition

On average, the number of benthic taxa increased over time in both control and sedimented flumes, but there were no statistical differences (Fig. 4a). However, a significant reduction in the total abundance of macroinvertebrates was observed as a consequence of sediment addition which was evident at both T1 and T2 (Fig. 4b). No statistical differences were recorded in functional richness by sediment treatment or time (Fig. 4c). Functional dispersion (Fig. 4d) was slightly higher in the sedimented flume than the control with values increasing over time, but these differences were not statistically significant (Table 2). Functional evenness (Fig. 4e) significantly increased over time in both treatments and was always slightly higher in the control than sedimented flumes (Table 2).

**Table 2:** Summary results of the linear regression models for the taxonomic and functional metrics of benthic and drifting macroinvertebrates. Significant values are in bold

Dataset	Community metric	Factor	Chi-square / F-value	*p*-value
Benthic	Taxon richness	Treatment	0.020	0.887
		Time	5.142	**0.023**
		TreatmentXTime	0.028	0.868
	Total density	Treatment	5.508	**0.019**
		Time	0.119	0.730
		TreatmentXTime	0.445	0.505
	Func. richness	Treatment	0.154	0.705
		Time	0.406	0.542
		TreatmentXTime	0.195	0.670
	Func. dispersion	Treatment	2.249	0.172
		Time	0.951	0.358
		TreatmentXTime	0.106	0.753
	Func. evenness	Treatment	1.767	0.220
		Time	6.945	**0.029**
		TreatmentXTime	0.301	0.598
Drift	Taxon richness	Treatment	0.582	0.446
		Time	5.302	**0.021**
		TreatmentXTime	1.076	0.299
	Drift density	Treatment	0.345	0.557
		Time	10.961	** < 0.001**
		TreatmentXTime	0.937	0.333
	Func. richness	Treatment	1.880	0.208
		Time	31.546	** < 0.001**
		TreatmentXTime	1.876	0.208
	Func. dispersion	Treatment	0.013	0.912
		Time	1.749	0.223
		TreatmentXTime	0.034	0.858
	Func. evenness	Treatment	0.874	0.393
		Time	1.058	0.351
		TreatmentXTime	1.647	0.256

**Fig. 5:**
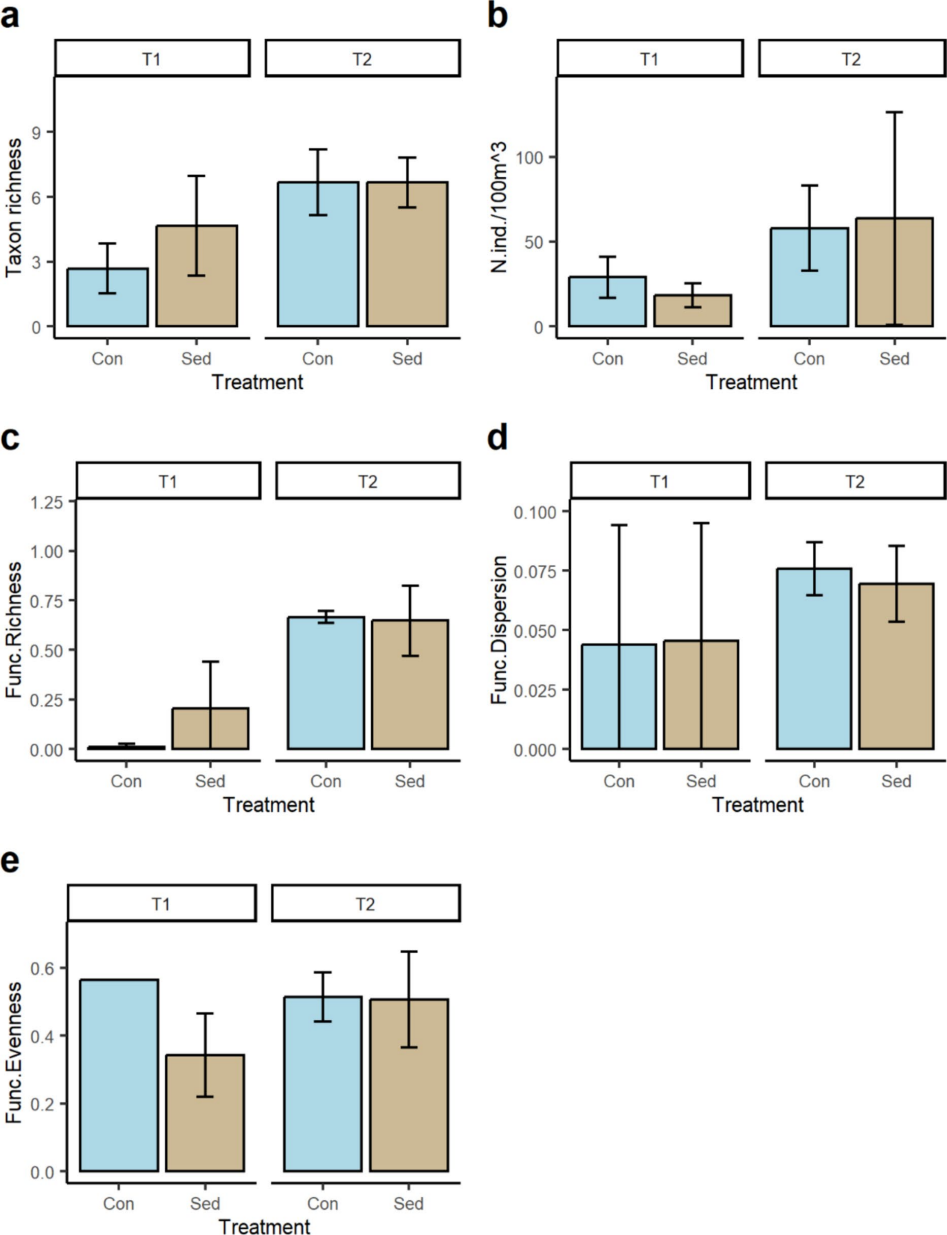
Error bars (± 1 SD) showing mean **a** taxon richness, **b** drift density, **c** functional richness, **d** functional dispersion, and **e** functional evenness of the drifting macroinvertebrate communities by sediment treatment on each sampling occasion. T1 = 24–48 h following sediment addition, and T2 = 2 weeks following sediment addition

The average taxon richness of drifting macroinvertebrates was slightly higher in the sedimented flumes than control flumes at T1 with the number of taxa significantly greater in both flumes at T2 displaying similar values (Fig. 5a; Table 2). In addition, total drift density (Fig. 5b) and functional richness (Fig. 5c) significantly increased from T1 to T2 but did not differ by sediment treatment (Table 2). No statistical differences in functional dispersion (Fig. 5d) and functional evenness (Fig. 5e) over time or by treatment were observed (Table 2).

## Discussion

The aim of this flume-based experiment was to assess the direct impacts of fine sediment addition on taxonomic and functional facets of Alpine macroinvertebrate communities. The study incorporated both benthic and drifting macroinvertebrates, thus providing simultaneous observations and evidence regarding direct fine sediment effects for stream macroinvertebrates (but see Connolly & Pearson, 2007; Conroy et al. 2016; Larsen & Ormerod, 2010). We observed major effects of fine sediment addition in relation to the taxonomic composition and heterogeneity of both benthic and drifting macroinvertebrates. Benthic macroinvertebrate taxonomic composition differed between control and sedimented flumes and has been a widely reported consequence of enhanced fine sediment deposition (Folegot et al. 2021; Williams & Etfa, 2024). The taxonomic heterogeneity of benthic communities increased over time (i.e. from T1 to T2) in control flumes; however, the opposite trend (homogenisation of communities) was observed in sedimented flumes. These findings indicate that the fine sediment addition affected the temporal trajectories of change of the macroinvertebrate communities as a potential consequence of the altered bed substrate conditions. Analyses of the components of beta diversity supported this evidence. Nestedness was the main driver of beta-diversity variation among benthic communities and is most likely due to the colonization process over time in both flume treatments. However, greater contributions of turnover (taxa replacement) were found to structure the control communities two weeks into the experiment (T2) compared to the sedimented communities and the control communities 1 day after sediment addition. These findings suggest that, in addition to the increased number of taxa colonizing over time, the substrate conditions of the control flumes also facilitated taxa replacement over time as a possible consequence of enhanced heterogeneity of biotic interactions and niche-based processes (Castro et al. 2025). In contrast, the dominant component of temporal variation in beta diversity of sedimented communities (i.e. from T1 to T2), was mostly driven by the increase of small number of generalist taxa (e.g. Lumbriculidae, Lumbricidae), suggesting a possible filter effect of sedimentation on the colonizing macroinvertebrates. Similar results were observed in previous studies, where nestedness was found to be the main mechanism structuring sedimented macroinvertebrate communities relative to control communities in mountainous (Buendia et al. 2013) and alpine river systems (Mathers et al. 2022). Mathers et al. (2022) also reported a strengthening effect of fine sedimentation effects on alpine communities temporally not observed in a lowland stream.

Six benthic taxa, namely *Protonemura* sp., *Amphinemura* sp., Hydropsychidae, Elmidae, Empididae, and Psychodidae, were more abundant in the sedimented flumes, acting as indicator taxa. *Nemoura* sp., *Habroleptoides* sp., and Chironomidae were classified as specialist taxa within control flumes. These findings partially support previous research on the taxon-specific tolerance of macroinvertebrates to fine sediment (Larsen et al. 2011; Doretto et al. 2018a). Psychodidae and Leptophlebiidae were classified as sediment-tolerant and sediment-sensitive taxa respectively in the current study. In marked contrast to our results, Extence et al. (2013) classified *Protonemura* sp., *Amphinemura* sp., and Hydropsychidae as sediment-sensitive in UK rivers. In several laboratory experiments, the behavioural response and survival rate of *Hydropsyche* species exposed to different doses of fine sand has been examined (Runde & Hellenthal, 2000; Wood et al. 2005; Conroy et al. 2018). These studies reported that *Hydropsyche* species were able to remain “buried-alive” when exposed to medium to coarse sand, while only the finest fraction of sediment caused increased mortality and drift rates (Runde & Hellenthal, 2000). Wood et al. (2005) and Conroy et al. (2018) found that *Hydropsyche* species are able to excavate to avoid the burial by fine sediment but this ability depends on the fine sediment grain size and thickness of the sediment layer. Moreover, previous studies showed that the microhabitat preferences and distribution within different substrates of Hydropsychidae may vary during their life cycle (Rutherford & Makay, 1985; Ficsór & Csabai, 2021). It should be noted that the fine sediment used in this experiment consisted of relatively coarse (0.5–1.5 mm) sterile play sand. As a result, the direct impacts associated with the finest sediment fraction (< 0.05 mm) fractions could not be determined. For example, suspended particles may exert an abrasive action on the surface of fragile and sensitive anatomical parts, such as eyes and respiratory structures, or they may clog gills or the mouthparts, especially for filter-feeding species including *Hydropsyche* (Bilotta & Brazier, 2008; McKenzie et al. 2020). This may partially explain the divergent response of some macroinvertebrate taxa reported in this study compared to previous studies. The mineralogical nature of the fine sediment addition may also lead to varying responses with some taxa responding more preferentially to the inorganic component and others the organic, while others display no difference in sensitivity (Mckenzie et al. 2025; Mathers et al. 2024).

The contrasting responses of taxa may also be associated with context dependency, with a number of studies now documenting that the effects of fine sediment are unlikely to be similar across river types and geographical regions (Mckenzie et al. 2024; Mathers et al. 2022; 2024). Indeed, some taxa display behavioural plasticity and differential responses to burial by fine sediment (Conroy et al. 2018). Community responses to fine sedimentation have also been shown to be dependent on the landscape setting—upland or lowland (Connolly & Pearson, 2007; Mathers et al. 2022) as well as species-specific responses (Conroy et al. 2018). Environmental context is also likely to mediate the implications of fine sediment in terms of the physical processes of fine sediment infiltration. Riverbeds comprised of predominately large substrates (e.g. cobbles and larger gravel particles) are likely to support large interstitial pore space and therefore may display less severe impacts associated with fine sediment deposition events (Dubuis & Cesear, 2023) as the open interstitial pore space may be retained to a certain degree even with fine sediment infiltration. In contrast, for finer grained substrates, fine sediment deposition and infiltration may lead to the development of clogs which is likely to limit interstitial habitat availability and reduce habitat heterogeneity and quality. In our study, the experimental substrates mimicked the upper headwaters of the Po River which is comprised predominately of small boulders and medium to large cobbles, while the gravel and sand classes were composed of the coarse fractions (31.5–8 mm = 35.6%; 4–8 mm = 29.8%; 4–1 mm = 26.2%; < 1 mm = 8.5%; personal data). Therefore, it is likely that the fine sediment addition event acted like a habitat disturbance, although the effect was not as great as anticipated or as marked as that observed in lowland fine-grained rivers with lower fine sediment additions (5 kg m^2^; Larsen & Ormerod, 2010; Wood et al. 2016). This may explain the weak differences in the community metrics observed in this study for both benthic and drifting invertebrates. All but one of the taxonomic and functional community metrics varied over time, but not by sediment treatment. Only the total density of benthic macroinvertebrates was found to be significantly lower in sedimented flumes than control flumes on both sampling occasions. It could be hypothesized that the fine sediment addition and deposition decreased the abundance of benthic macroinvertebrates as a consequence of, at least partial, substrate colmation and reduced pore spaces as reported in both field studies (Vasconcelos & Melo, 2008; Mathers et al. 2022) and mesocosm experiments (Shaw & Richardson, 2001). Our findings suggest that future research should examine the effects of grain size and volume of fine sediment added, the substrate framework that receives the fine sediment, the macroinvertebrate taxa present as well as role of season and landscape setting (e.g. uplands vs lowland) on macroinvertebrate communities (Mathers et al. 2023). In addition, as our results were obtained from a relatively short experiment, future studies should examine the long-term response of macroinvertebrates to fine sediment pulses and deposition.

In this study, Chironomidae were the dominant macroinvertebrate taxon of benthic communities and fine sediment addition reduced their overall abundance. Highly variable responses of the dipteran family Chironomidae to fine sediment have been widely documented in the scientific literature (Couceiro et al. 2010; Leitner et al. 2015). This probably reflects the systematic resolution, as previously noted (Zweig & Rabeni, 2001; Larsen et al. 2009), because Chironomidae is a very large family encompassing many genera and species. As a consequence, it is expected that greater insights regarding the sensitivity and tolerance to fine sediment may be obtained at finer taxonomic resolutions. For instance, Angradi (1999) found that the relative abundance of subfamilies Orthocladiinae and Chironominae increased and decreased with higher fine sediment loads, respectively. When developing a stressor-specific biomonitoring index for assessing the siltation-driven impacts for macroinvertebrates in the UK, Extence et al. (2013, 2017) excluded Chironomidae larvae from the index calculation owing to the inability of scoring this family with a single coherent sensitivity score. Similarly, Gieswein et al. (2019) concluded that Chironomidae as a family were unable to indicate riverbed fine sediment content because of inconsistent and weak responses. Furthermore, the time of sampling may also have some influence on taxa specific responses to fine sediment addition with two genera of Chironomidae (*Chironomus* and *Stictochironomus)* being identified as indicator species in summer and autumn for sand habitats but not in spring (Mathers et al. 2023).

Unlike benthic assemblages, the taxonomic composition of drifting invertebrates demonstrated temporal differences but no direct effects of fine sediment addition. This was particularly evident for sedimented communities which, from T1 to T2, demonstrated a significant increase in the taxonomic heterogeneity of drifting macroinvertebrates relative to control communities. Moreover, analyses of beta diversity and its components revealed that compositional changes in drifting invertebrates were largely driven by nestedness. These results suggest that the drift rates of macroinvertebrates in this experiment were most likely affected by colonization dynamics over time and the large pore sizes of the experimental substrate likely mitigated the effect of fine sediment addition on resultant drift behaviour. Another possible reason is that by using 0.5–1.5-mm sterile play sand without the finer fraction, the concentration of suspended solids was likely not affected and this, in turn, may have affected the drifting behaviour of macroinvertebrates which could be more susceptible to suspended sediment effects (but see Larsen & Ormerod, 2010). Nevertheless, the highest contribution of taxa replacement to total beta diversity between control and sedimented communities was observed at T1, suggesting that the sediment pulse was, at least partially, enough to have an immediate effect on some drifting taxa. It is reasonable to hypothesize that the fine sediment deposition altered the substrate conditions sufficiently enough to stimulate the drift of new colonizing taxa that did not find suitable microhabitats in the sedimented flumes. This likely explains the increased heterogeneous composition of drifting invertebrates in the sedimented flumes observed at T2 and may suggest a drift behavioural response to the altered substrate conditions.

In an in situ manipulative study, Connolly & Pearson (2007) installed PVC flumes (3.60 × 0.30 × 0.15 m) in a tropical small order stream (Camp Creek, Paluma, Australia) and evaluated the differences in the drift density of macroinvertebrates following fine sediment addition (i.e. 750 g of clay suspended in 20 l of water and siphoned in the mesocosms by using a 10 mm plastic tube). Similar to our experiment, the authors did not record differences in the drift densities between control and sedimented flumes (Connolly & Pearson, 2007). In a flume-based experiment in Ireland, Conroy et al. (2016) added varying amounts of fine sediment (diameter < 1 mm) to achieve eight different levels of sediment deposition (100–0%) and, while no differences in total drift density were observed among treatments, increased drift of Heptageniidae was generally observed in channels with higher levels of sediment addition. In contrast, in a field experiment, Culp et al. (1986) added sand (0.5–0.2 mm) to a riffle mesohabitat and recorded that the sediment addition increased drift rates. Clearly, the implications of fine sediment addition on drift are not consistent and highlight the need for taxon-specific assessments and context-dependent evaluations of invertebrate drift response to fine sediment pulses (deposition and suspension).

In a review on the experimental design and approaches with flume-shaped mesocosms, Menczelesz et al. (2020) reported that the number of published papers increased over time from 1975 to 2018. The authors also reported that the growing application was not evenly distributed across continents, with some geographical areas and settings being under-represented and less-equipped to undertake flume-based facilities (Menczelesz et al. 2020). When considering the field of river ecology, the adoption of experimental flumes has increased in recent decades insofar as these mesocosms allow us to better evaluate the pure and synergic effects of multiple stressors, especially on benthic communities (Bækkelie et al. 2017; Pacheco et al. 2022; Rosenkranz et al. 2023). Nevertheless, flumes are still poorly used to evaluate the ecological impacts of fine sediment, even if anthropogenic-induced siltation is globally recognized as one of the main sources of impairment for river ecosystems (Gupta et al. 2023). To our knowledge, this is the first flume-based study dealing with the combined response of benthic and drifting macroinvertebrates to fine sediment addition in an Alpine setting. Given the growing awareness of context specific effects of stressors, the use of flume-based investigations in a variety of geographical settings will aid our mechanistic understanding of fine sediment. The results of this study contribute to disentangling the effects of fine sediment on Alpine macroinvertebrate communities from additive and synergic effects due to co-occurring factors and to the multi-country comparisons of the ecological impacts of fine sediment deposition in rivers.

## Supplementary Information

Below is the link to the electronic supplementary material.

Supplementary file1 (DOCX 533 kb)

## Data Availability

Enquiries about data availability should be directed to the authors.
