# Supplementary material for: Taxonomic and functional responses of benthic and drifting macroinvertebrates to fine sediment deposition: evidence from an alpine flume-based experiment
Source: Hydrobiologia. 2025 Nov 13;853(5):1527–46. doi: 10.1007/s10750-025-06014-w (PMC12855233; doi:10.1007/s10750-025-06014-w)
Supplement: Supplementary file 1 — Supplementary file1 (DOCX 533 kb) [file 10750_2025_6014_MOESM1_ESM.docx]

**Taxonomic and functional responses of benthic and drifting macroinvertebrates to fine sediment deposition: evidence from an alpine flume-based experiment**

**Supplementary Information**


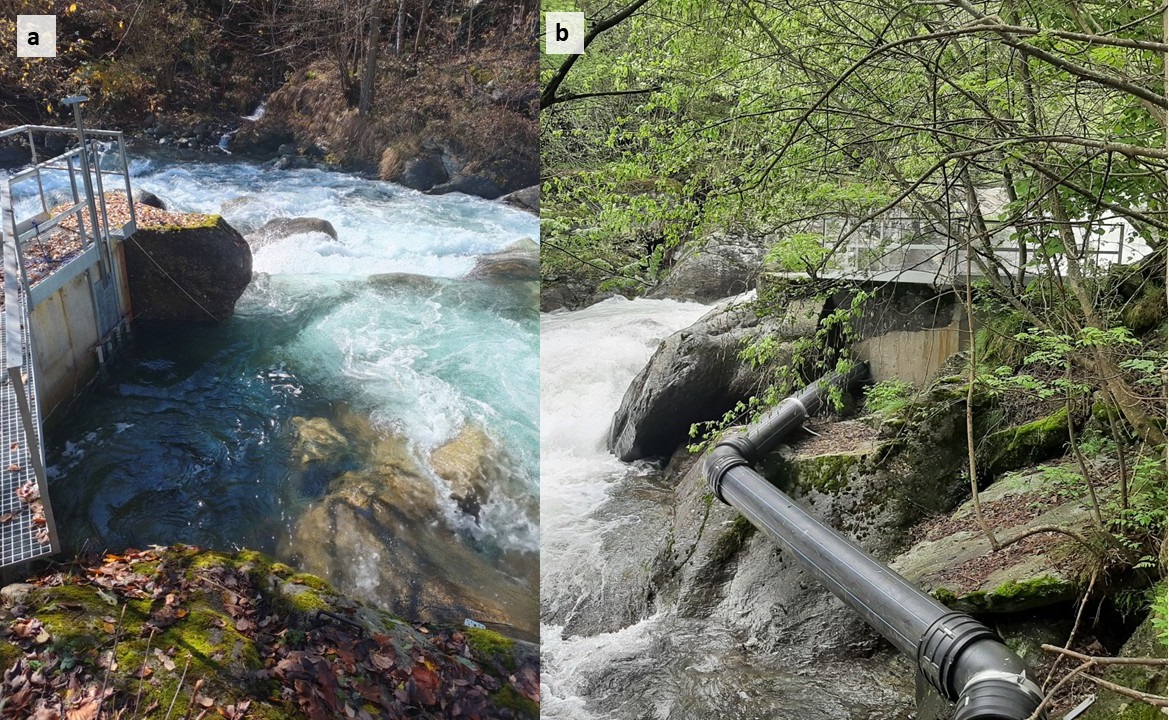


Figure S1. Photographs of: (a) the small water diversion on the Po River (around 40 m upstream of the flumes), and (b) the pipe that delivers water to the flumes from the Po River. Photographs were taken in different seasons.

Table S1. Physical and chemical parameters measured in the Po River reach near the flume facility over the experiment.

| **Date (dd/mm/yyyy)** | **Time** | **Dissolved oxygen**  **(mg/L)** | **Conductivity (µS/cm)** | **Temperature (°C)** | **pH** | **Mean water velocity (m/s)** | | **Mean water depth (cm)** | |
| --- | --- | --- | --- | --- | --- | --- | --- | --- | --- |
|  |  |  |  |  |  | **Con** | **Sed** | **Con** | **Sed** |
| 19/04/2023 | T0 | 10.99 | 178.30 | 7.00 | 7.98 | 0.18 | 0.14 | 10.56 | 10.61 |
| 09/05/2023 | T1 | 10.28 | 132.90 | 9.50 | 7.53 | 0.16 | 0.17 | 9.67 | 10.28 |
| 23/05/2023 | T2 | 10.70 | 88.00 | 9.20 | 7.46 | 0.13 | 0.12 | 11.17 | 10.33 |

Table S2. List of benthic macroinvertebrate taxa found in the flumes (control and sedimented) on each sampling occasion (T1 and T2). Symbols represent the ranges of total abundance: + = 1-10 individuals, ++ = 11-100 individuals, +++ = more than 100 individuals.

| **Benthic** | **T1** | | **T2** | |
| --- | --- | --- | --- | --- |
| **Taxon** | **Control flumes** | **Sedimented flumes** | **Control flumes** | **Sedimented flumes** |
| *Leuctra* | + | ++ | + | + |
| *Isoperla* | + | + |  | + |
| *Protonemura* |  | + | + | + |
| *Amphinemura* | +++ | +++ | +++ | +++ |
| *Nemoura* | + |  | + | + |
| *Ecdyonurus* | ++ | + | + | ++ |
| *Rhithrogena* |  | + | + |  |
| *Serratella* |  |  | + | + |
| *Baetis* | ++ | ++ | ++ | ++ |
| *Epeorus* |  |  |  | + |
| *Habroleptoides* | + |  | + | + |
| Hydropsychidae |  | + | + | + |
| Limnephilidae | + | + | + | + |
| Philopotamidae |  |  | + |  |
| Rhyacophilidae | + |  |  | + |
| Lepidostomatidae |  |  |  | + |
| Scirtidae |  | + |  | + |
| Hydrophilidae |  | + |  |  |
| Hydraenidae | + |  |  |  |
| Elmidae | ++ | ++ | ++ | ++ |
| Chironomidae | +++ | +++ | +++ | +++ |
| Psychodidae | ++ | ++ | ++ | ++ |
| Simuliidae | + |  | + | + |
| Blephariceridae | + |  | + |  |
| Anthomyiidae |  |  | + |  |
| Limoniidae |  | + | + | + |
| Athericidae | + | + |  | + |
| Empididae | + | + | + | ++ |
| Ceratopogonidae | + | + | ++ | + |
| Dixidae |  | + | + | + |
| Lumbriculidae | + | + | ++ | ++ |
| Lumbricidae |  | + | + | + |
| Naididae | + | + |  |  |
| Osmylidae |  |  |  | + |
| *Sialis* |  |  | + |  |

Table S3. List of drifting macroinvertebrate taxa found in the flumes (control and sedimented) on each sampling occasion (T1 and T2). Symbols represent the ranges of total abundance: + = 1-10 individuals, ++ = 11-100 individuals, +++ = more than 100 individuals.

| **Drift** | **T1** | | **T2** | |
| --- | --- | --- | --- | --- |
| **Taxon** | **Control flumes** | **Sedimented flumes** | **Control flumes** | **Sedimented flumes** |
| *Leuctra* |  | + | + | + |
| *Protonemura* | + | + | + |  |
| *Amphinemura* | + | + | + | + |
| *Nemoura* |  |  |  | + |
| *Ecdyonurus* |  |  | + | + |
| *Baetis* |  | + | + | + |
| *Serratella* |  | + |  |  |
| Rhyacophilidae |  |  |  | + |
| Chironomidae | +++ | +++ | +++ | +++ |
| Psychodidae | + | + | ++ | + |
| Anthomyiidae |  |  | + |  |
| Empididae |  |  | + | + |
| Dixidae |  | + |  |  |
| Elmidae |  |  |  | + |
| Lumbricidae |  |  |  | + |
| Lumbriculidae |  |  | ++ | ++ |
| Naididae |  | + |  |  |
| Nematoda |  | + | ++ | ++ |
